# Correlative Analysis of Tumor-Informed Circulating Tumor DNA (ctDNA) and the Survival Outcomes of Patients with Pancreatic Adenocarcinoma

**DOI:** 10.3390/biomedicines13051124

**Published:** 2025-05-06

**Authors:** Yuqi Zhang, Abdullah Esmail, Hala Hassanain, Vikramjit Dhillon, Waseem Abdelrahim, Ebtesam Al-Najjar, Bayan Khasawneh, Maen Abdelrahim

**Affiliations:** 1 Section of GI Oncology, Houston Methodist Neal Cancer Center, Houston Methodist Hospital, Houston, TX 77094, USA; 2 Michael E. DeBakey HS for Health Professions, Houston, TX 77030, USA

**Keywords:** pancreatic ductal adenocarcinoma, PDAC, circulating tumor DNA, minimal residual disease, MRD, recurrence, progression-free survival, overall survival, tumor-informed assay, biomarker, prognostic marker, CA19-9, adjuvant chemotherapy, non-invasive monitoring, liquid biopsy, personalized treatment, surveillance

## Abstract

**Background:** Pancreatic ductal adenocarcinoma (PDAC) is a highly aggressive cancer with poor prognosis due to late-stage diagnosis, limited surgical resectability, and frequent recurrence. Traditional biomarkers like CA19-9 and imaging techniques often fail to detect minimal residual disease (MRD) or early recurrence. Circulating tumor DNA (ctDNA) is a promising non-invasive biomarker that may provide early detection of disease recurrence, offering a potential improvement in patient management. This study aimed to assess the utility of ctDNA as a prognostic tool for PDAC patients, specifically in predicting recurrence and overall survival (OS). **Methods:** This retrospective study analyzed data from 39 PDAC patients who underwent surgery and were monitored for ctDNA levels using Signatera™, a tumor-informed multiplex PCR next-generation sequencing assay. Blood samples were collected both preoperatively and postoperatively, and ctDNA levels were measured to detect MRD. The sensitivity, specificity, positive predictive value (PPV), and negative predictive value (NPV) of ctDNA were compared with CA19-9 in detecting disease recurrence. Clinical outcomes, including progression-free survival (PFS) and OS, were evaluated in relation to ctDNA status. **Results:** Among 39 patients, 153 plasma samples were analyzed, with 17 patients testing positive for ctDNA. Sensitivity of ctDNA in detecting relapse was 91%, compared to 83% for CA19-9, with combined testing reaching 98% sensitivity. ctDNA positivity was associated with significantly shorter OS and PFS, with patients testing negative for ctDNA having a median OS of 37.6 months versus 13.4 months in ctDNA-positive patients (*p* = 0.003). The median time from ctDNA positivity to imaging-confirmed relapse was 81 days. Positive ctDNA was also linked to higher rates of lymphovascular invasion and positive surgical margins, highlighting the aggressive nature of the disease in these patients. **Conclusions:** CtDNA is a highly sensitive and specific biomarker for detecting MRD and predicting recurrence in PDAC patients, offering superior performance over CA19-9. Positive ctDNA results were associated with worse prognosis, including shorter OS and PFS, and may help guide treatment decisions. These findings suggest that ctDNA could be a valuable tool for personalized management in PDAC, though further prospective studies are needed to validate its clinical role in treatment stratification.

## 1. Introduction

In 2019, there were an estimated 56,770 individuals in the United States who received a new diagnosis of pancreatic cancer, and roughly 46,000 died from the disease [1]. While the mortality rates of stomach and colorectal cancers have been decreasing in the last twenty years, there has been no reduction in mortality rates for pancreatic cancer [1,2,3]. Currently, pancreatic cancer accounts for approximately 3% of all cancer diagnoses and 7% of cancer-related fatalities [4]. It is expected that by 2030, pancreatic cancer will become the second leading cause of cancer-related mortality [5].

Approximately 80–90% of pancreatic malignancies are categorized as pancreatic ductal adenocarcinomas (PDACs) [2,3,6,7,8]. A primary factor contributing to the high death rate of PDACs is the prevalence of advanced-stage disease upon diagnosis in most patients. At the time of diagnosis, only a small percentage, specifically 15–20% of patients, are deemed suitable for surgical intervention [9]. In addition, the prognosis for patients who undergo surgery with negative margins is still unfavorable, with a 5-year survival rate ranging from only 10–25% and a median survival of 10–20 months [10]. The National Comprehensive Cancer Network recommends 6-month adjuvant chemotherapy for curative intent after surgery for patients with resectable PDAC [11,12]. Yet, more than 75% of patients experience recurrence following surgery [13]. The current 5-year survival rate for PDAC is 12% in general but drops to 3% for individuals with metastatic disease [4,14]. However, the National Comprehensive Cancer Network (NCCN) panel and other specialized pancreatic cancer centers currently recommend that patients who are very likely to have early signs of metastasis receive neoadjuvant therapy six months prior to any surgical intervention [15]. The American Society of Clinical Oncology (ASCO) guidelines also recommend neoadjuvant therapy for patients with resectable PDAC who cannot undergo immediate surgery [16,17].

Optimization of treatment depends on identification of high-risk patients who are more likely to recur or progress and would benefit most from neoadjuvant and adjuvant systemic therapy. Recent prospective trials have shown that adjuvant treatments are suitable for all patients with resectable or borderline resectable PDAC [15,18,19]. However, the implementation of adjuvant treatments may not be feasible for all patients undergoing surgical resection, primarily due to the high incidence of postoperative complications. Moreover, it is frequently essential to reduce the dose of chemotherapy in patients who have had surgery, potentially resulting in reduced efficacy of the treatment. Neoadjuvant treatments may be a better choice from this point of view because they can be given to a larger group of patients who are planning to have curative procedures and because they have a higher rate of therapeutic efficacy [20]. Currently, clinicians monitor diseases using a combination of clinical symptoms, a tumor marker known as cancer antigen 19-9 (CA 19-9), and computed tomography (CT) or magnetic resonance imaging (MRI) [21]. Still, CA-19-9 levels can rise in both cancer and noncancerous conditions, like biliary inflammation or blockage, which makes it a biomarker that is not very specific [22,23]. Yet traditional imaging techniques are often not sensitive enough to detect minimum residual disease (MRD), defined as the presence of residual cancer cells undetectable by routine imaging or lab tests but potentially leading to relapse [24,25].

Circulating tumor DNA (ctDNA) is a blood biomarker capable of detecting disease recurrence prior detection on imaging studies [26,27,28]. Liquid biopsy is an emerging technique that enables the collection of tumor material that is circulating in the body without the need for invasive procedures, and its efficacy is being investigated in various forms of gastrointestinal malignancies [29]. It is expected to offer both practical and theoretical advantages over current diagnostic standards [30,31]. The use of cfDNA, ctDNA, tumor-derived exosomes, and circulating tumor cells (CTCs) in PDAC has attracted much attention lately [32,33]. Studies in various cancers have shown that ctDNA is a dependable technique for detecting MRD and informing treatment decisions [34,35,36]. In this study, we tracked ctDNA levels over time using Signatera^TM^, a special multiplex PCR next-generation sequencing (NGS) assay. The objective was to verify ctDNA as a disease-monitoring tool that can assess effectiveness of treatment during the perioperative phase, and predict the likelihood of recurrence during surveillance. Furthermore, we aim to confirm the utility of ctDNA as a dependable indicator of recurrence that can be used to improve patient risk stratification throughout treatment.

## 2. Methodology

### 2.1. Study Cohort and Sample Collection

This study retrospectively analyzed real-world data of individuals with PDAC and ampullary carcinoma. Data from commercial ctDNA testing conducted between December 2019 to November 2023 were used from a selection of 39 patients diagnosed with PDAC. In the period between June 2020 and December 2023, blood samples were obtained from these patients and stored in a biobank. Each sample underwent ctDNA testing utilizing the tumor-informed assay Signatera^TM^. The exclusion criteria encompassed patients who did not possess Signatera results, lacked complete and validated clinical data or follow-up, had other histologic subtypes such as pancreatic neuroendocrine tumors, or did not provide informed consent. The individuals who were eligible for the investigation were those who had a confirmed diagnosis of PDAC or ampullary carcinoma (N = 1) as well as longitudinal data on ctDNA and disease-free survival (DFS). Plasma samples were collected both before and after the surgical procedure. The samples were examined for ctDNA. In addition to that, ctDNA analysis was carried out throughout the monitoring period. Initial ctDNA was generally obtained 14 to 534 days after surgery, with a median of 75 days followed by every 1 to 3 months thereafter until death or last follow up.

Clinical and pathological data of every patient were collected. Both the biobank patient samples and the commercial patient samples, which were treated to perioperative care, displayed homogeneity in their biological characteristics. All of the patients, with the exception of the biobank cohort, were treated and monitored according to the expert opinion of the attending physician. Prior to the information regarding the clinical data being disclosed, the statistical analysis plan for ctDNA was developed. Anonymization was performed on the data before it was evaluated. Patient consent was obtained prior to sample collection.

### 2.2. Personalized mPCR-Based NGS Assay for ctDNA Detection

Signatera^TM^, a multiplex PCR NGS method, was used to detect and measure tissue-specific DNA. Whole exome sequencing was performed on cell-free DNA from blood samples and tumor blocks from the same patients. To account for germline mutations, both tumor tissue and matched normal tissue (germline) from each patient are sequenced to identify patient-specific mutations and then, using this information, a custom, personalized assay is designed. This assay then targets tumor-specific clonal mutations, filtering out germline mutations. Universal libraries were produced using specialized adapters and end repair, A-tailing, and ligation processes. Libraries were then processed through barcoding, consolidation, mPCR amplification, and sequencing with NGS technology. Plasma samples were considered ctDNA-positive if they displayed a minimum of two different alterations. The quantity of ctDNA was calculated by determining the average number of tumor molecules (MTM) present in one milliliter of plasma.

## 3. Results

### 3.1. Patient Characteristics

Plasma samples (n = 153) from patients (N = 39) with PDAC (18 females vs. 21 males) were obtained. The median age at diagnosis was 67 years old (Range: 41–83). Baseline patient characteristics between patients with ctDNA that was initially negative (ctDNA (−)) (N = 22) and initially positive (ctDNA (+)) (N = 17) were comparable (see Table 1).

A larger portion of ctDNA (−) patients had resectable disease (18 of 21) compared to the ctDNA (+) group (11 out of 17). Of those who underwent surgical resection, a higher percentage of ctDNA (−) patients had negative margins (94% compared to 64%) compared to ctDNA (+) patients. Lymphovascular invasion (LVI) was higher in ctDNA (+) patients (91% compared to 28% in ctDNA (−) patients) in those who underwent resection.

### 3.2. ctDNA as Detection for MRD

**Figure 1:**
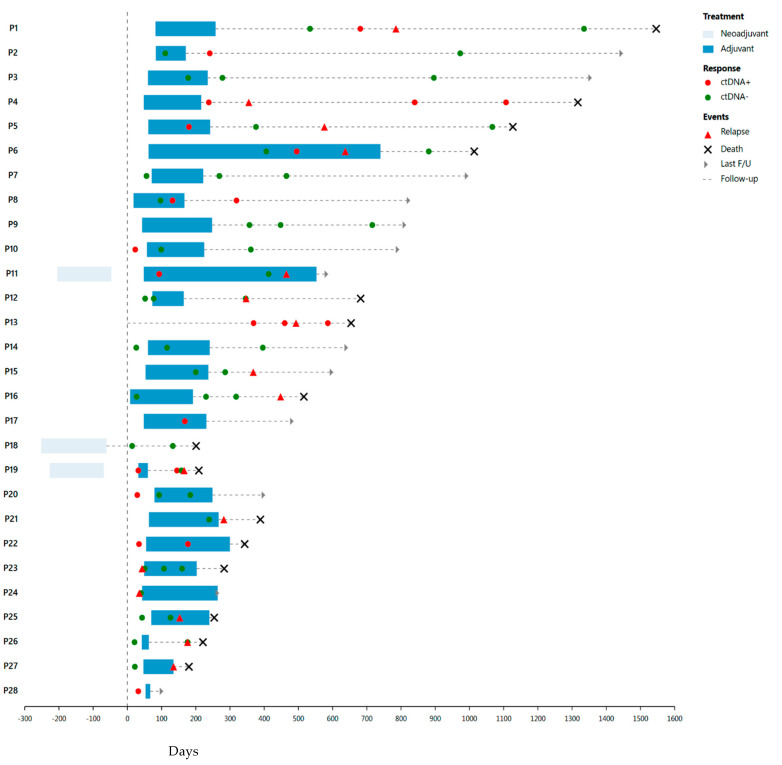
**ctDNA for MRD detection after surgery.** Patients are monitored before and after surgery (on Day 0) for relapse as confirmed on imaging with ctDNA and followed until death, data cut off time, or being lost to follow up.

Of the 29 patients who underwent surgery for PDAC, 15 (52%) had ctDNA collected within the MRD window (defined as within 3 months after resection), with 9 of the 15 (60%) having positive ctDNA in the MRD window. All of the nine patients had confirmed progression on later imaging (detection rate 100%). Twenty-six of the twenty-nine received adjuvant chemotherapy with either gemcitabine and abraxane or FOLFIRINOX. Two of the ctDNA (−) patients also received neoadjuvant chemotherapy in addition to adjuvant therapy (see Figure 1).

The sensitivities, specificities, and positive and negative predictive values of the ctDNA test compared to CA19-9 are listed in Table 2. Briefly, in this context, positive predictive value is a measure of how accurately a positive ctDNA result indicates residual PDAC.

Sensitivity and negative predictive value (NPV) of ctDNA in detection of residual or progressive disease as confirmed by CT imaging were higher at 91% (CI 72–99%) and 87% (CI 60–98%), compared to that of CA19-9 (83% (CI 61–95%) and 75% (CI 48–93%)), respectively. Meanwhile, specificities were very similar between ctDNA and CA19-9 testing (81% (CI 54–96%) vs. 80% (CI 52–96%)). Notably, combining the two tests, sensitivity and specificity increased to 98% and 96%, with corresponding increases in PPV and NPV (97% and 98%).

### 3.3. ctDNA as a Prognostic Marker

Of fifteen patients with ctDNA collected within the MRD window after surgery, eight had positive ctDNA while seven had negative ctDNA. Fourteen of the fifteen (including all but one ctDNA (−) patient who had received neoadjuvant chemotherapy) received adjuvant chemotherapy. Of patients who underwent resection for PDAC, 14 had at least one instance of ctDNA positivity associated with disease relapses or progression, with a median time between positive ctDNA and CT-confirmed relapse/progression of 81 days (Figure 1). This highlights the utility of ctDNA in predicting disease progression.

**Figure 2:**
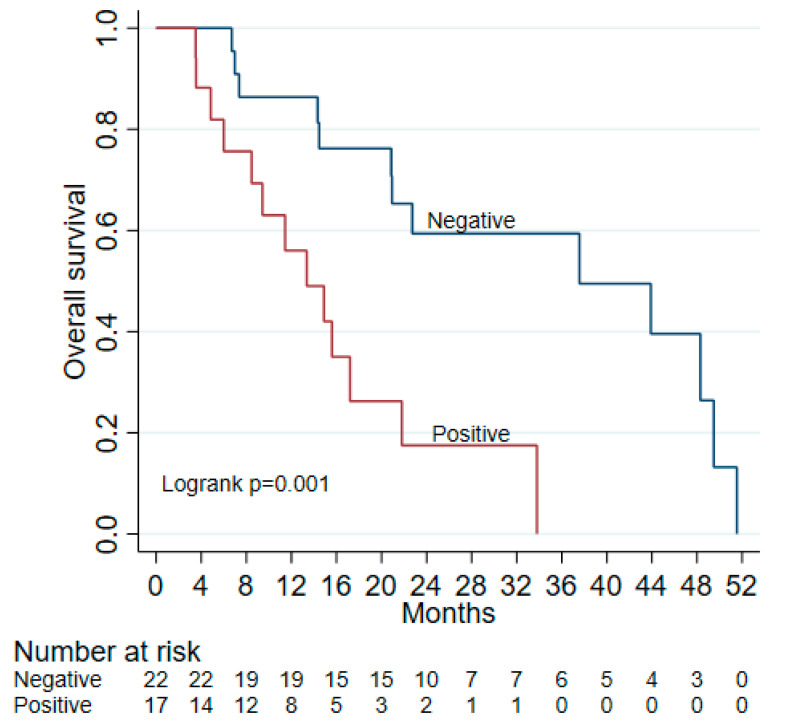
Overall survival by initial ctDNA.

Median follow up was significantly different between ctDNA (−) and ctDNA (+) groups, at 22 and 11 months, respectively (*p* = 0.009). In those with initially negative ctDNA (including two with positive surgical margins), seven had progression after a median of 12.3 months (range 5.5–37.4 months), compared to mPFS of 8.7 months (range 1.1–26.2 months) in ctDNA (+) patients. Most patients who received adjuvant chemotherapy progressed while on treatment (see Figure 1). The median overall survival (OS) for ctDNA (−) patients was significantly longer at 37.6 (95% CI: 20.9–49.5) months, compared to ctDNA (+) patients (13.4 (95% CI: 6–21.8) months) (see Figure 2). Overall, a positive initial ctDNA is associated with higher mortality compared to a negative ctDNA test (HR: 3.97, 95% CI: 1.61–9.80, *p* = 0.003).

## 4. Discussion

Previous studies have shown that positive ctDNA is associated with shorter PFS and OS in patients who undergo resection for pancreatic cancer [26,27,28]. For example, one study reported a median survival of 13.6 months vs. 27.6 months in patients with detectable vs. no detectable ctDNA, which is similar to the findings from our study [35]. Compared to other surveillance tools (e.g., CA19-9 and ctDNA detection using a panel of commonly mutated genes), tumor-informed ctDNA has been proposed to be a more sensitive and specific detection method with a high prognostic value [27,28]. In this study, using tumor-informed ctDNA samples from patients at our institution, we found a 24.2-month difference in OS between ctDNA (−) and ctDNA (+) patients, which further supports the use of ctDNA as a prognostic biomarker in PDAC.

As adjuvant chemotherapy is indicated for localized pancreatic cancer, tumor-informed ctDNA can be collected within the MRD window as a prognostic tool and beyond the MRD window to monitor for recurrence. In a meta-analysis by Lee et al. [37], positive ctDNA at baseline and postoperatively is associated with decreased overall survival (HR 2.27 and HR 3.66, respectively) and a higher risk of disease recurrence [37]. In the current study, patients were tested for ctDNA within and beyond the MRD window. Positive ctDNA was not associated with age, sex, or location of the pancreatic tumor. However, a lack of ctDNA detection was associated with negative margins and the lack of lymphovascular invasion (LVI), which are, in themselves, positive prognostic factors. Given the higher percentage of high-risk features in our study—positive margins (36% vs. 5%) and lymphovascular invasion (91% vs. 32%)—in patients with initial ctDNA+ disease, adjuvant therapy may be especially warranted for this patient population. Indeed, data from this as well as other studies supports ctDNA testing in tandem with other prognostic factors to predict patient outcomes and guide management decisions.

Monitoring for recurrence is uniquely challenging for PDAC compared to other gastrointestinal malignancies such as colorectal carcinoma and cholangiocarcinoma given the lack of sensitive and specific tumor markers. Given the more aggressive nature of the disease, a more sensitive yet accessible test has been long sought-after. As a surveillance test, we show that ctDNA has a higher sensitivity than the more commonly used CA19-9. Sensitivity and specificity increase when both are used to monitor biochemical disease progression even before detection by imaging. Considering this, ctDNA alone or in conjunction with CA19-9 can be used to more reliably track treatment response. A previous study demonstrated that ctDNA levels decreased significantly after treatment initiation but increased at the time of progression, providing a lead time over CA19-9 [38]. As such, ctDNA may be a powerful tool to guide treatment decisions in all phases of PDAC including localized, borderline-resectable, and metastatic disease. Persistent ctDNA positivity indicates aggressive disease biology that may require alternative therapies, while a negative result could lead to treatment de-escalation.

The study has a few considerations, including the cohort size and its retrospective, single-institutional design. While only a subset of patients underwent ctDNA testing within the MRD window, the findings suggest that a positive ctDNA result, regardless of when it is found with regard to diagnosis or surgery, is strongly associated with a worse prognosis. This can help guide clinicians toward closer surveillance and more aggressive treatment options for high-risk patients. The absence of preoperative ctDNA samples in resected patients is another consideration, though future studies may explore the prognostic value of changes in ctDNA levels postoperatively. Despite these factors, this study provides valuable insights into ctDNA’s role in PDAC management, supporting its potential as a robust biomarker for disease monitoring and prognosis.

Randomized prospective trials are needed to assess the value of ctDNA in the prognosis and risk stratification for neoadjuvant and adjuvant management in PDAC. Currently, the CASPER trial (NCT05853198) seeks to evaluate ctDNA as a marker of surgical futility in a single arm of patients with operable PDAC, with an endpoint of relapse within 2 years of surgery. In addition, the FRENCH.MRD.PDAC is a French trial yet to begin recruitment, that aims to determine disease-free survival (DFS) and ctDNA detection status after curative surgery and adjuvant chemotherapy. Overall, ctDNA promises to be a valuable tool for personalized management of PDAC patients.

## Figures and Tables

**Table 1:** Patient characteristics.

	ctDNA (−)	ctDNA (+)
Male sex	12 (55%)	9 (53%)
Median age	65 (48–82)	68 (40–83)
**Site of tumor**		
Head	17 (77%)	13 (76%)
Body/tail	5 (23%)	4 (24%)
Underwent resection	19 (86%)	11 (65%)
Margins clear on resection	18 (95%)	7 (64%)
Lymphovascular invasion present	6 (32%)	10 (91%)

**Table 2:** Sensitivity and specificity of ctDNA.

	ctDNA	CA19-9	Combined
Sensitivity	0.913	0.826	0.98
Specificity	0.813	0.8	0.96
Positive predictive value (PPV)	0.875	0.863	0.97
Negative predictive value (NPV)	0.867	0.75	0.98

## Data Availability

The data from this study that support our results are available upon request from the corresponding author, Maen Abdelrahim.

## References

[1] Siegel R.L., Miller K.D., Jemal A. (2019). Cancer statistics, 2019. CA Cancer J. Clin..

[2] Abdelrahim M., Esmail A., Kasi A., Esnaola N.F., Xiu J., Baca Y., Weinberg B.A. (2024). Comparative molecular profiling of pancreatic ductal adenocarcinoma of the head versus body and tail. NPJ Precis. Oncol..

[3] Bugazia D., Al-Najjar E., Esmail A., Abdelrahim S., Abboud K., Abdelrahim A., Umoru G., Rayyan H.A., Abudayyeh A., Al Moustafa A.E. (2024). Pancreatic ductal adenocarcinoma: The latest on diagnosis, molecular profiling, and systemic treatments. Front. Oncol..

[4] (2024). Pancreatic Cancer Statistics.

[5] Rahib L., Smith B.D., Aizenberg R., Rosenzweig A.B., Fleshman J.M., Matrisian L.M. (2014). Projecting cancer incidence and deaths to 2030: The unexpected burden of thyroid, liver, and pancreas cancers in the United States. Cancer Res..

[6] Ducreux M., Cuhna A.S., Caramella C., Hollebecque A., Burtin P., Goéré D., Seufferlein T., Haustermans K., Van Laethem J.L., Conroy T. (2015). Cancer of the pancreas: ESMO Clinical Practice Guidelines for diagnosis, treatment and follow-up. Ann. Oncol..

[7] Badheeb M., Abdelrahim A., Esmail A., Umoru G., Abboud K., Al-Najjar E., Rasheed G., Alkhulaifawi M., Abudayyeh A., Abdelrahim M. (2022). Pancreatic tumorigenesis: Precursors, genetic risk factors and screening. Curr. Oncol..

[8] Esmail A., Badheeb M., Abdelrahim M. (2023). Pancreatic Tumorigenesis: Precursors, Genetic Risk Factors and Screening. Pancreatic Cancer-Updates in Pathogenesis, Diagnosis and Therapies.

[9] Adamska A., Domenichini A., Falasca M. (2017). Pancreatic Ductal Adenocarcinoma: Current and Evolving Therapies. Int. J. Mol. Sci..

[10] Oettle H., Neuhaus P., Hochhaus A., Hartmann J.T., Gellert K., Ridwelski K., Niedergethmann M., Zülke C., Fahlke J., Arning M.B. (2013). Adjuvant chemotherapy with gemcitabine and long-term outcomes among patients with resected pancreatic cancer: The CONKO-001 randomized trial. JAMA.

[11] Kwaśniewska D., Fudalej M., Nurzyński P., Badowska-Kozakiewicz A., Czerw A., Cipora E., Sygit K., Bandurska E., Deptała A. (2023). How A Patient with Resectable or Borderline Resectable Pancreatic Cancer should Be Treated-A Comprehensive Review. Cancers.

[12] Chikhladze S., Lederer A.K., Kousoulas L., Reinmuth M., Sick O., Fichtner-Feigl S., Wittel U.A. (2019). Adjuvant chemotherapy after surgery for pancreatic ductal adenocarcinoma: Retrospective real-life data. World J. Surg. Oncol..

[13] Lee J.C., Ahn S., Cho I.K., Lee J., Kim J., Hwang J.H. (2018). Management of recurrent pancreatic cancer after surgical resection: A protocol for systematic review, evidence mapping and meta-analysis. BMJ Open.

[14] Park W., Chawla A., O’Reilly E.M. (2021). Pancreatic Cancer: A Review. JAMA.

[15] Tempero M.A., Malafa M.P., Al-Hawary M., Behrman S.W., Benson A.B., Cardin D.B., Chiorean E.G., Chung V., Czito B., Del Chiaro M. (2021). Pancreatic Adenocarcinoma, Version 2.2021, NCCN Clinical Practice Guidelines in Oncology. J. Natl. Compr. Canc. Netw..

[16] Aoki T., Mori S., Kubota K. (2024). Neoadjuvant and Adjuvant Chemotherapy for Pancreatic Adenocarcinoma: Literature Review and Our Experience of NAC-GS. Cancers.

[17] Vivarelli M., Mocchegiani F., Nicolini D., Vecchi A., Conte G., Dalla Bona E., Rossi R., Benedetti Cacciaguerra A. (2022). Neoadjuvant Treatment in Resectable Pancreatic Cancer. Is It Time for Pushing on It?. Front. Oncol..

[18] Sugumar K., Hue J.J., De La Serna S., Rothermel L.D., Ocuin L.M., Hardacre J.M., Ammori J.B., Winter J.M. (2021). The importance of time-to-adjuvant treatment on survival with pancreatic cancer: A systematic review and meta-analysis. Cancer Rep..

[19] Khorana A.A., McKernin S.E., Berlin J., Hong T.S., Maitra A., Moravek C., Mumber M., Schulick R., Zeh H.J., Katz M.H.G. (2019). Potentially Curable Pancreatic Adenocarcinoma: ASCO Clinical Practice Guideline Update. J. Clin. Oncol..

[20] Smaglo B.G. (2023). Role for Neoadjuvant Systemic Therapy for Potentially Resectable Pancreatic Cancer. Cancers.

[21] Lee T., Teng T.Z.J., Shelat V.G. (2020). Carbohydrate antigen 19-9—Tumor marker: Past, present, and future. World J. Gastrointest. Surg..

[22] Kim S., Park B.K., Seo J.H., Choi J., Choi J.W., Lee C.K., Chung J.B., Park Y., Kim D.W. (2020). Carbohydrate antigen 19-9 elevation without evidence of malignant or pancreatobiliary diseases. Sci. Rep..

[23] Tsen A., Barbara M., Rosenkranz L. (2018). Dilemma of elevated CA 19-9 in biliary pathology. Pancreatology.

[24] Campos N.M.F., Almeida V., Curvo Semedo L. (2022). Peritoneal disease: Key imaging findings that help in the differential diagnosis. Br. J. Radiol..

[25] Dong Q., Chen C., Hu Y., Zhang W., Yang X., Qi Y., Zhu C., Chen X., Shen X., Ji W. (2023). Clinical application of molecular residual disease detection by circulation tumor DNA in solid cancers and a comparison of technologies: Review article. Cancer Biol. Ther..

[26] Watanabe F., Suzuki K., Aizawa H., Endo Y., Takayama Y., Kakizawa N., Kato T., Noda H., Rikiyama T. (2023). Circulating tumor DNA in molecular assessment feasibly predicts early progression of pancreatic cancer that cannot be identified via initial imaging. Sci. Rep..

[27] Botta G.P., Abdelrahim M., Aushev V.N., Esmail A., Drummond B., Sharma S., Kalashnikova E., Hook N., Chandana S.R., Tejani M.A. (2022). Association of personalized and tumor-informed ctDNA with patient survival outcomes in pancreatic adenocarcinoma. J. Clin. Oncol..

[28] Botta G.P., Abdelrahim M., Drengler R.L., Aushev V.N., Esmail A., Laliotis G., Brewer C.M., George G.V., Abbate S.M., Chandana S.R. (2024). Association of personalized and tumor-informed ctDNA with patient survival outcomes in pancreatic adenocarcinoma. Oncologist.

[29] Marrugo-Ramírez J., Mir M., Samitier J. (2018). Blood-Based Cancer Biomarkers in Liquid Biopsy: A Promising Non-Invasive Alternative to Tissue Biopsy. Int. J. Mol. Sci..

[30] Crowley E., Di Nicolantonio F., Loupakis F., Bardelli A. (2013). Liquid biopsy: Monitoring cancer-genetics in the blood. Nat. Rev. Clin. Oncol..

[31] Vymetalkova V., Cervena K., Bartu L., Vodicka P. (2018). Circulating Cell-Free DNA and Colorectal Cancer: A Systematic Review. Int. J. Mol. Sci..

[32] Merker J.D., Oxnard G.R., Compton C., Diehn M., Hurley P., Lazar A.J., Lindeman N., Lockwood C.M., Rai A.J., Schilsky R.L. (2018). Circulating Tumor DNA Analysis in Patients With Cancer: American Society of Clinical Oncology and College of American Pathologists Joint Review. J. Clin. Oncol..

[33] Samandari M., Julia M.G., Rice A., Chronopoulos A., Del Rio Hernandez A.E. (2018). Liquid biopsies for management of pancreatic cancer. Transl. Res. J. Lab. Clin. Med..

[34] Patel H., Okamura R., Fanta P., Patel C., Lanman R.B., Raymond V.M., Kato S., Kurzrock R. (2019). Clinical correlates of blood-derived circulating tumor DNA in pancreatic cancer. J. Hematol. Oncol..

[35] Hadano N., Murakami Y., Uemura K., Hashimoto Y., Kondo N., Nakagawa N., Sueda T., Hiyama E. (2016). Prognostic value of circulating tumour DNA in patients undergoing curative resection for pancreatic cancer. Br. J. Cancer.

[36] Grunvald M.W., Jacobson R.A., Kuzel T.M., Pappas S.G., Masood A. (2020). Current Status of Circulating Tumor DNA Liquid Biopsy in Pancreatic Cancer. Int. J. Mol. Sci..

[37] Lee J.S., Rhee T.M., Pietrasz D., Bachet J.B., Laurent-Puig P., Kong S.Y., Takai E., Yachida S., Shibata T., Lee J.W. (2019). Circulating tumor DNA as a prognostic indicator in resectable pancreatic ductal adenocarcinoma: A systematic review and meta-analysis. Sci. Rep..

[38] Lapin M., Edland K.H., Tjensvoll K., Oltedal S., Austdal M., Garresori H., Rozenholc Y., Gilje B., Nordgård O. (2023). Comprehensive ctDNA Measurements Improve Prediction of Clinical Outcomes and Enable Dynamic Tracking of Disease Progression in Advanced Pancreatic Cancer. Clin. Cancer Res..

